# Speed matters: fast pace 10-metre walking test is superior to normal pace in predicting gait recovery following ventriculoperitoneal shunt insertion in normal pressure hydrocephalus

**DOI:** 10.1007/s00701-026-06783-w

**Published:** 2026-02-02

**Authors:** Christoph Wiest, Daoud Chaudhry, Saniya Mediratta, Emalee Burrows, Matthew Deehan, Simon Thompson, Lewis Thorne, Laurence Watkins, Ahmed K. Toma

**Affiliations:** 1 Victor Horsley Department of Neurosurgery, National Hospital for Neurology and Neurosurgery, Queen Square, London, UK; 2 Queen Square Institute of Neurology, University College London, London, UK; 3 Department of Neurosurgery, King’s College Hospital, London, UK

**Keywords:** Idiopathic normal pressure hydrocephalus, 10 m walking test, Ventriculoperitoneal shunt, Extended lumbar drainage

## Abstract

**Purpose:**

Idiopathic normal pressure hydrocephalus (iNPH) is characterised by Hakim’s tetrade comprising gait, balance, cognitive and urinary disturbance. As gait deteriorates early, 10-m walking tests (10MWT) before and after lumbar tap or extended lumbar drainage tests have been used to identify patients who may benefit from permanent cerebrospinal fluid diversion in the form of a ventriculoperitoneal (VP) shunt. Whether 10MWT should be performed at fast or normal pace to best predict benefit from shunting has been unclear so far.

**Methods:**

We included 125 iNPH patients into a retrospective, longitudinal, single-centre cohort study and performed 10MWT before and after 72-h lumbar drainage, immediately after VP shunt insertion and at the 6-month, 1-year, 2-year, 3-year, 5-year and 8-year marks postoperatively.

**Results:**

We found that time and step count improvements of normal and fast 10MWT before and after lumbar drainage were maintained in the first two to three years postoperatively. Furthermore, fast pace 10MWT time and step count better predicted postoperative gait improvement than normal pace 10MWT. Early responders of fast gait measures (walking pace improved by ≥ 0.1 m/s or step count improvement > 10% after lumbar drainage) were 3.91 (pace) and 6.29 (steps) times more likely to benefit from surgery as opposed to 2.64 (pace) and 1.93 (steps) times for normal walking pace.

**Conclusions:**

Our study suggests that the 10MWT should be performed at fast pace (maximum speed), and when normal and fast pace results are contradictory, the fast pace outcome should take priority.

## Introduction

Idiopathic normal pressure hydrocephalus (iNPH) is a potentially reversible cause of gait and balance disturbances, cognitive decline, and urinary incontinence in older adults [1]. Among its core symptoms, gait impairment is typically the earliest and most responsive to treatment, making it a focus of both diagnosis and long-term outcome assessment [11, 13]. In clinical practice, temporary cerebrospinal fluid (CSF) diversion, such as through CSF tap tests or extended lumbar drainage, is used to identify patients who are likely to benefit from permanent CSF diversion via ventriculoperitoneal (VP) shunting [5, 12, 15]. However, the predictive value of various clinical assessments performed after temporary CSF diversion remains uncertain.

Walking tests, particularly the 10-m walking test (10MWT), are commonly used to quantify gait changes [3]. These are often administered at both self-selected (normal pace) and maximum (fast pace) walking speeds, capturing different aspects of gait control and reserve [6]. While normal-speed walking may reflect everyday functioning, fast walking may be more sensitive to subtle deficits in gait automaticity and executive control, domains frequently affected in iNPH [2]. In clinical practice, contradictory 10MWT results, with improvement at fast pace but deterioration at normal pace or vice versa, complicate clinical decision-making. Whether early improvement in fast or normal pace walking tests after temporary CSF drainage more accurately predicts long-term postoperative outcomes has not been systematically tested [8]. This study aims to directly compare the predictive value of fast versus normal pace 10MWT, measured after 72-h lumbar drainage, in forecasting long-term gait improvement following VP shunt surgery.

## Methods

### Patient selection and consent

We conducted a retrospective longitudinal cohort study of iNPH patients treated at a single tertiary neurosurgical centre between 2013 and 2023. Patients with iNPH who underwent extended 72-h lumbar drainage and VP shunt insertion at the National Hospital for Neurology and Neurosurgery, London UK, were included. Routinely collected anonymised data were collected with University College London Hospitals NHS Foundation Trust service evaluation approval in accordance with the Declaration of Helsinki. According to institutional and national research ethics guidelines, formal ethical approval and individual patient consent were not required. Human Ethics and Consent to Participate declarations: not applicable. Patients were selected by an interdisciplinary team comprising neurosurgeons, neurologists, neuropsychiatrists and specialist hydrocephalus nurses.

### Clinical testing

Patients performed standardised 10MWT in an unobstructed environment at nine time points: before and after 72-h elective lumbar drainage, immediately after VP shunt insertion, and at follow-up visits after 6 months, 1 year, 2 years, 3 years, 5 years, and 8 years. At each time point, patients first completed a normal pace 10-m walk, where patients were asked to walk in their comfortable pace, as if for example they are walking to the shops, followed by a fast pace walk, where patients were asked to walk as fast as they could. The time taken and the number of steps to cover 10 m were recorded for both conditions. Patient numbers for each condition and time point are detailed in Table 1.

**Table 1:** Patient numbers included in all conditions

	Fast WT Time	Normal Speed WT Time	Fast WT Steps	Normal Speed WT Steps
preLD	106	119	100	110
postLD	106	119	100	110
post shunt	10	12	10	12
6 m	63	75	57	66
1y	63	67	58	62
2y	44	46	40	42
3y	20	25	19	22
5y	14	13	13	13
8y	9	9	8	7

*WT* Walking Test, *preLD* before lumbar drainage, *postLD* after lumbar drainage, *post shunt*  after shunt insertion, *6 m* = 6 months after shunt insertion, *1y-8y* = 1–8 years after shunt insertion

### Statistics

**Fig. 1:**
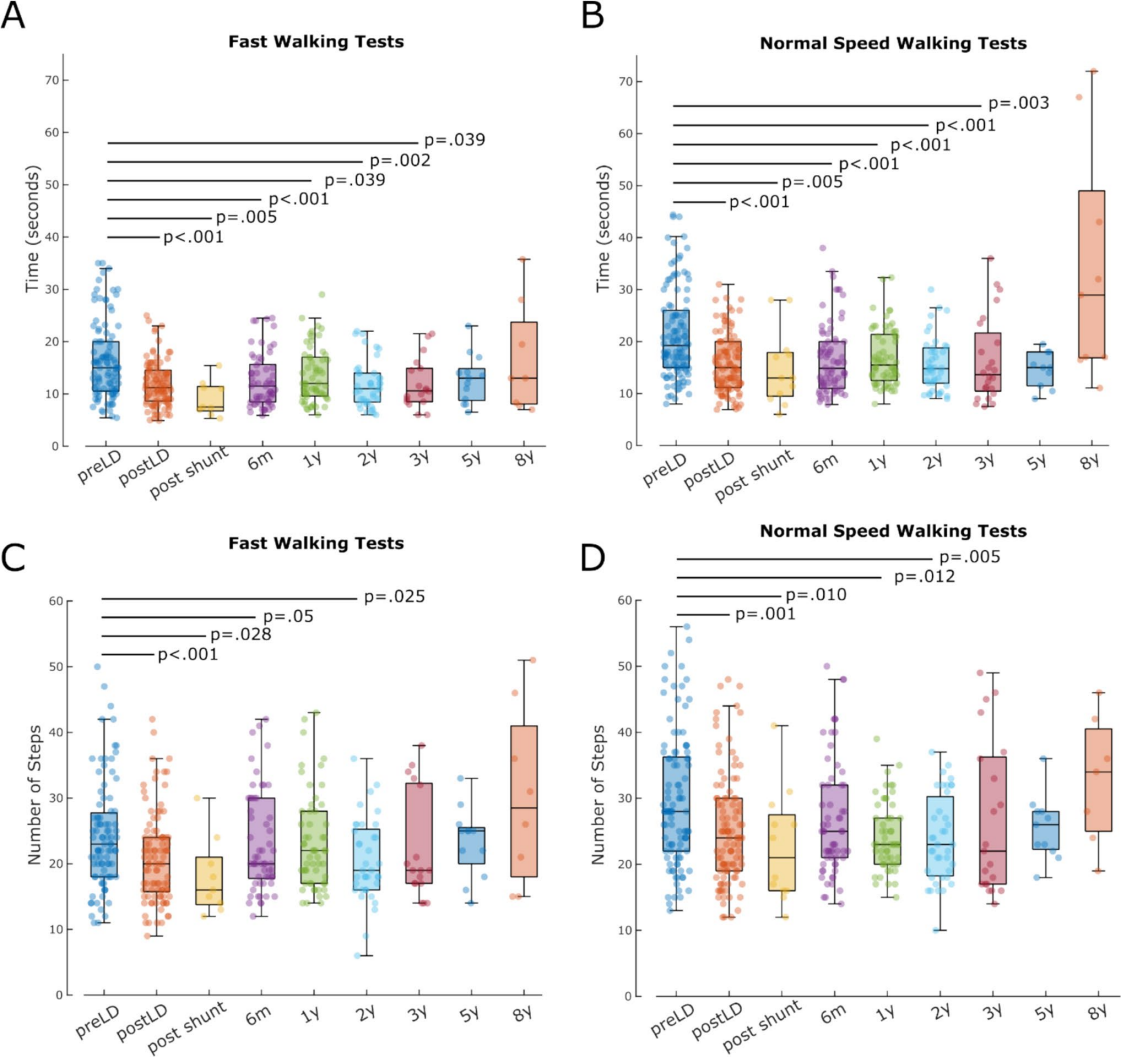
Time (**A**, **B**) and number of steps (**C**, **D**) of fast (**A**, **C**) and normal speed (**B**, **D**) 10-m walking tests decrease following 72-h lumbar drainage and remain below baseline until the 2-year follow up. preLD = before 72-h lumbar drainage, postLD = after 72-h lumbar drainage, post shunt = following shunt insertion, 6 m = 6 months after shunt insertion, 1-8y = 1–8 years after shunt insertion

The statistical analyses were conducted using custom-written scripts in MATLAB (MathWorks). As samples in Fig. 1 were non-normally distributed we used a two-sided Wilcoxon rank sum test to compare walking test times and step counts between follow-ups and preoperative baseline.

### Area under the curve (AUC)-based analysis

All participants who had follow-up after 6 months or 1 year were included in this part. We used walking pace and step counts before and after extended lumbar drainage to calculate early improvement and the values at the respective follow-ups for postoperative improvement. Where follow-up was performed at both 6 and 12 months, only the best performance of both follow-ups was included. A previous study suggested walking pace improvement of at least 0.1 m/s as clinically meaningful [10] and we used this threshold to binarise preoperative candidates as likely responders (pace improvement ≥ 0.1 m/s and step count improvement ≥ 10%) and likely non-responders. We subsequently performed a Receiver Operating Characteristic (ROC) analysis using MATLAB’s *perfcurve* function, and computed Youden’s J index and sensitivity and specificity at the optimal cut-off. Finally, we used a generalised linear regression model with the formula ‘PostopResponder ~ PreopResponder’ to get an estimate of how much more likely an early responder (after lumbar drainage) is to benefit from surgery.

## Results

### Walking performance improves following lumbar drainage and is maintained after VP shunt insertion

125 patients (87 male, 38 female) with iNPH, average age at referral was 74.90 ± 6.65 (mean ± SD) years, were included in the study. First, we aimed to reproduce previous studies confirming that walking test outcomes improve after 72-h lumbar drainage and that these benefits are maintained following shunt insertion. Our data showed that the time taken for fast 10MWT was significantly reduced after lumbar drainage (*p* < 0.001; Fig. 1A). This effect was confirmed by a smaller cohort immediately post shunt insertion (*p* = 0.005) and by more representative cohorts (see Table 1) after six months (*p* < 0.001), one year (*p* = 0.039), two years (*p* = 0.002) and three years (*p* = 0.039). Similarly, the number of steps needed during fast pace 10MWT significantly decreased post lumbar drainage (*p* < 0.001; Fig. 1C) which was maintained immediately following shunt insertion (*p* = 0.028), after six months (*p* = 0.05) and two years (*p* = 0.025).

Similar trends were observed for normal pace walking tests. The time taken significantly decreased after extended lumbar drainage (*p* < 0.001; Fig. 1B) and this effect was sustained immediately after surgery (*p* = 0.005), after six months (*p* < 0.001), one year (*p* < 0.001), two years (*p* < 0.001) and three years (*p* = 0.003). Accordingly, the number of steps needed for 10 m at normal pace was significantly lower post lumbar drainage (*p* = 0.001; Fig. 1D) and this remained below baseline levels immediately after shunt surgery (*p* = 0.010), after one year (*p* = 0.012) and after two years (*p* = 0.005).

### Fast pace walking tests are a better predictor of post-shunt walking improvement than normal speed walking tests

**Fig. 2:**
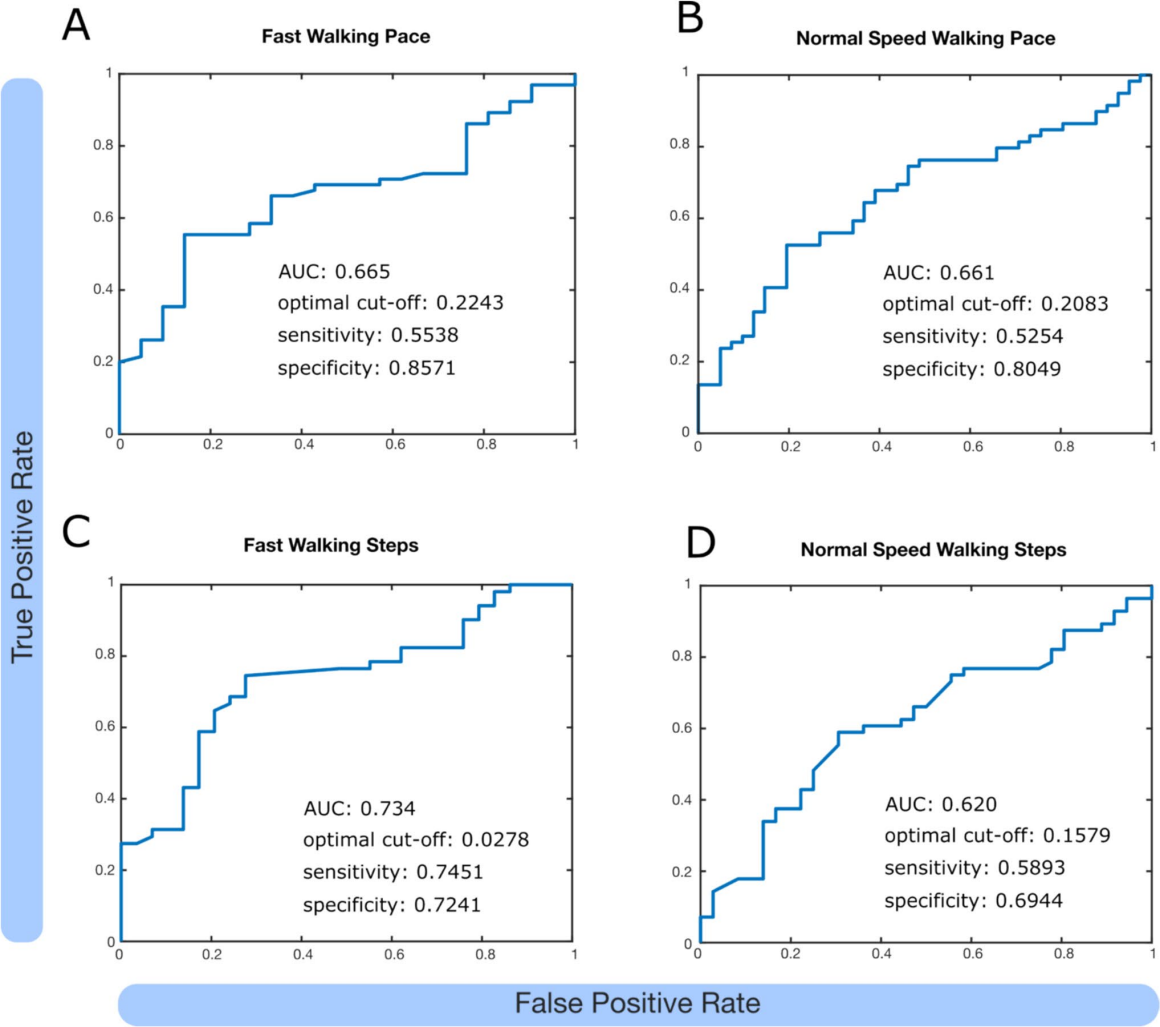
We defined participants with walking pace improvement of at least 0.1 m/s (**A**, **B**) or step count reduction of 10% (**C**, **D**) post lumbar drainage as early responders and performed a ROC analysis (see Methods for further details). AUC: area under the curve, optimal cut off as defined by Youden’s J index, sensitivity and specificity at optimal cut off

To evaluate whether fast or normal pace 10MWT post lumbar drainage more accurately predict long-term postoperative gait improvement after permanent CSF diversion, we performed a ROC analysis. Using postoperative fast walking pace gain as the outcome and a binary indicator of preoperative improvement ≥ 0.1 m/s as the classifier, the model yielded an AUC of 0.665. The ROC analysis identified an optimal cutoff of 0.224 m/s preoperative improvement (Youden’s J), which achieved a sensitivity of 0.554 and a specificity of 0.857 for predicting postoperative responders (Fig. 2A). To evaluate whether preoperative gait responders were more likely to demonstrate a clinically meaningful postoperative fast walking improvement, we fitted a logistic regression model with postoperative responder status as the dependent variable and preoperative responder status (≥ 0.1 m/s improvement) as the predictor. Preoperative responders had significantly higher odds of postoperative improvement (β = 1.36, SE = 0.53, *p* = 0.010), corresponding to an odds ratio of 3.91.

For normal pace walking tests, the model yielded an AUC of 0.661 with an optimal cutoff of 0.2083 m/s preoperative improvement, which achieved a sensitivity of 0.5254 and specificity of 0.8049 for predicting postoperative responders (Fig. 2B). Again, preoperative responders had higher odds of postoperative improvement (β = 0.96, SE = 0.42, *p* = 0.021), corresponding to an odds ratio of 2.64.

For fast walking step counts, the ROC analysis yielded an AUC of 0.734 with an optimal cutoff of 27.8% improvement, which achieved a sensitivity of 0.7451 and a specificity of 0.7241 (Fig. 2C). Preoperative responders had higher odds of post-shunt step improvement (β = 1.84, SE = 0.53, *p* = 0.0005), corresponding to an odds ratio of 6.29.

Lastly, for normal pace step counts, the model yielded an AUC of 0.620 with an optimal cutoff at 15.79% improvement, which achieved a sensitivity of 0.5893 and a specificity of 0.6944 (Fig. 2D). Preoperative responders did not have significantly higher odds of postoperative step improvement (β = 0.66, SE = 0.43, *p* = 0.13), yielding an odds ratio of 1.93.

This suggests that early gains in fast walking pace and step count strongly predict postoperative response, whereas normal pace metrics show weaker or inconsistent predictive value.

## Discussion

This study provides evidence that early gait improvements after extended lumbar drainage, particularly in fast pace walking tests, are strong predictors of long-term postoperative gait outcomes in patients with iNPH undergoing VP shunting. Both normal and fast pace walking speeds improved significantly after 72-h CSF diversion and remained below baseline following shunt insertion (Fig. 1). In the AUC-based analysis, we demonstrate that patients who improved by at least 0.1 m/s after lumbar drainage were 3.91 times more likely to achieve postoperative clinically meaningful improvement in fast walking as compared to 2.64 times more likely in normal speed. Patients whose step count in fast walks improved by 10% or more after lumbar drainage, were 6.29 times more likely to benefit from VP shunt insertion as compared to normal walks where the model did not yield a significant benefit (OR = 1.93).

Our findings support and extend previous work demonstrating that temporary CSF drainage via large-volume lumbar taps [4, 14, 16] or 72-h lumbar drainage [9, 17] can help identify shunt responders. The significant improvements observed in both walking time and step count after lumbar drainage confirm its value not just as a diagnostic tool, but also as a short-term therapeutic intervention. Of note, both walking time and step count were not significantly different from baseline beyond three years post surgery likely due to incomplete follow-up, patient attrition (Table 1) and disease progression.

A strength of this study lies in the direct comparison between fast and normal pace 10MWT after extended lumbar drainage, which to our knowledge has not previously been systematically evaluated in this context. A previous study compared fast with normal pace 10MWT after large volume CSF taps in a moderate cohort (*N* = 29) and found that fast pace walking tests had higher specificity and superior diagnostic performance [6], which is confirmed in our larger data set (Fig. 2). Although normal pace walking has traditionally been used to assess everyday functioning, our results suggest that fast pace walking tests may better capture latent gait deficits, likely reflecting reduced automaticity and executive dysfunction, both of which are core features of the iNPH phenotype [7]. This hypothesis is supported by prior work showing that dual-task or complex gait paradigms are more sensitive to deficits in frontal-subcortical circuits affected by NPH [2].

Our findings have several clinical implications. First, they reinforce the use of extended lumbar drainage as a valuable prognostic tool, especially when standardised walking tests are used before and after lumbar drainage. Second, they suggest that clinicians should consider incorporating fast pace walking assessments into routine iNPH evaluation, as these may reveal subtle impairments not apparent at normal pace. Third, in borderline cases with contradictory results of fast and normal pace walking tests, i.e. where normal pace deteriorates after ELD and fast pace improves, fast pace should take priority and the test should be considered positive.

Several limitations warrant consideration. This was a retrospective study from a single centre, and although patient numbers were high and follow-up extended up to 8 years, only a subset of patients were tested at each follow-up (Table 1). Furthermore, we only performed 10MWT concealing gait changes at longer distances. While other gait changes like decreased step height, widened stepping base, increased trunk sway and impaired walking balance were not recorded in our cohort, they likely impact speed and step count and are indirectly reflected. Although data were collected by multiple clinicians over a 10-year period, any inter-observer variability is expected to have balanced out over time. Moreover, while gait is the most responsive symptom to shunting, this study did not evaluate cognitive or urinary outcomes, which may follow different trajectories. Future work could explore whether fast walking tests also predict multidomain recovery or whether combining gait, cognitive, and radiological markers enhances predictive accuracy.

In conclusion, this study demonstrates that early improvement in fast pace 10MWT following lumbar drainage is a more sensitive and specific predictor of long-term gait improvement after VP shunt surgery in iNPH than normal pace walking tests. Particularly if fast and normal pace walking tests show conflicting results, outcomes of fast pace assessments should take priority for surgical decision making. These findings argue for a greater emphasis on dynamic, speed-sensitive gait measures in both diagnostic and prognostic assessment of iNPH.

## Data Availability

No datasets were generated or analysed during the current study.

## Abbreviations

10MWT: 10-metre walking tests
AUC: Area under the curve
CSF: Cerebrospinal fluid
ELD: Extended lumbar drainage
iNPH: Idiopathic normal pressure hydrocephalus
LD: Lumbar drainage
ROC: Receiver operating characteristic
VP: Ventriculoperitoneal
